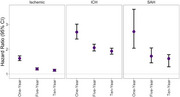# Stroke Risk Among Persons Diagnosed with Dementia:A Population‐Based Cohort Study in Denmark

**DOI:** 10.1002/alz70860_098291

**Published:** 2025-12-23

**Authors:** Holly C Elser, Erzsébet Horváth Puhó, Cecilia Hvitfeldt Fuglsang, Victor W. Henderson, Henrik Toft Sørensen

**Affiliations:** ^1^ Hospital of the University of Pennsylvania, Philadelphia, PA, USA; ^2^ Aarhus University, Aarhus, Denmark; ^3^ Stanford University School of Medicine, Stanford, CA, USA

## Abstract

**Background:**

Cognitive impairment is a common and well‐recognized consequence of stroke, and pre‐existing dementia is prevalent among stroke patients. However, research that examines stroke incidence among persons with an existing dementia diagnosis remains scarce with mixed findings‐to‐date.

**Method:**

This nationwide population‐based cohort study leverages routinely and prospectively collected data from Danish population‐based registries from 1996–2021. Persons with dementia with no prior history of stroke age ≥35 years were identified using diagnostic codes from the *International Classification of Diseases, Tenth Revision* from the Danish National Patient Registry. Persons diagnosed with dementia were matched on age, sex, and calendar year to members of the general population with no dementia diagnosis with a ratio of 3:1. Using Cox proportional hazards regression models, we computed hazard ratios (HRs) and 95% confidence intervals adjusted for matching variables for the association of dementia with ischemic stroke, intracerebral hemorrhage (ICH), and subarachnoid hemorrhage (SAH) with one, five, and ten years’ follow‐up after dementia diagnosis.

**Result:**

We identified 158,725 persons diagnosed with dementia and 476,170 matched persons. More than half of the study population was female (61%). Among persons diagnosed with dementia, there were 12,620 (8%) incident strokes diagnosed during follow‐up. The hazard of ischemic stroke was elevated among persons diagnosed with dementia compared to matched persons throughout the study period, with attenuated magnitude of association with longer follow‐up (one‐year: HR=1.63, 95%CI: 1.54,1.73; five‐year: 1.20, 95%CI: 1.16,1.25; ten‐year: HR=1.15, 95%CI: 1.11,1.19). We observed stronger associations of dementia diagnoses with ICH (one‐year: 2.69, 95%CI: 2.41,3.01; five‐year: HR=2.06, 95%CI: 1.93,2.20; ten‐year: HR=1.92, 95%CI: 1.80,2.04) and SAH (one‐year: HR=2.71, 95%CI: 2.04,3.61; five‐year: HR=1.72, 95%CI: 1.45,2.05; ten‐year: HR=1.52, 95%CI: 1.29,1.78) (Figure).

**Conclusion:**

In this population‐based cohort study, we observed persistently elevated stroke risk among persons diagnosed with dementia compared with matched persons. These results underscore the importance of aggressive management of vascular risk factors.